# Long-Term Influence of Endodontic Irrigants on In Vitro Dentin Biomimetic Remineralization

**DOI:** 10.3390/biomimetics11070473

**Published:** 2026-07-07

**Authors:** Paola Taddei, Michele Di Foggia, Andrea Spinelli, Maria Giovanna Gandolfi, Carlo Prati, Fausto Zamparini

**Affiliations:** 1 Biochemistry Unit, Department of Biomedical and Neuromotor Sciences, University of Bologna, Via Irnerio 48, 40126 Bologna, Italy; paola.taddei@unibo.it; 2 Endodontic Clinical Section, Unit of Odontostomatological Sciences, Department of Biomedical and Neuromotor Sciences, University of Bologna, Via San Vitale 59, 40126 Bologna, Italy; andrea.spinelli4@unibo.it (A.S.); carlo.prati@unibo.it (C.P.); fausto.zamparini2@unibo.it (F.Z.); 3 Laboratory of Biomaterials and Oral Pathology, Unit of Odontostomatological Sciences, Department of Biomedical and Neuromotor Sciences, University of Bologna, Via San Vitale 59, 40126 Bologna, Italy; mgiovanna.gandolfi@unibo.it

**Keywords:** irrigants, EDTA, citric acid, chlorhexidine, NaClO, dentin remineralization, apatite, vibrational IR spectroscopy

## Abstract

Endodontic irrigant solutions act as crucial pretreatment conditioning agents in dentin biomimetic remineralization, preparing the collagen scaffold for calcium phosphate infiltration and subsequent tooth structure reconstruction. In this study, root dentin discs were exposed for 10 min to five irrigant solutions: sodium hypochlorite (NaClO, 3%), EDTA (17%), citric acid (CA, 10%), chlorhexidine (CHX, 2%), and an innovative experimental formulation containing citric acid (7%) and surfactants. Samples were then aged in Hank’s Balanced Salt Solution (HBSS) at 37 °C for three months to simulate long-term clinical conditions. Physicochemical modifications of the collagen and apatite phases were assessed at each experimental stage using ATR-FTIR spectroscopy, with the A_CaP_/A_Amide I_ and A_870_/A_CaP_ absorbance ratios as markers of the degree of mineralization and apatite carbonate content, respectively. Results indicated that CHX- and EDTA-treated dentin exhibited the highest remineralization after ageing, while NaClO impeded remineralization due to collagen degradation. The experimental irrigant produced the most pronounced demineralization, followed by CA; however, it also facilitated significant remineralization, attributed to citrate–collagen binding and surfactant-enhanced apatite nucleation. NaClO selectively degraded collagen and increased apatite crystallinity; CA inhibited apatite nucleation through adsorbed citrate ions, and CHX and EDTA induced minimal alterations. These findings provide molecular-level evidence linking short-term irrigant effects to the long-term potential for dentin biomineralization, with direct implications for irrigant selection in regenerative endodontic protocols. It should be noted that this study was conducted on dentin discs obtained from a single tooth; all findings should therefore be regarded as preliminary and require confirmation in studies with larger, biologically independent sample sizes.

## 1. Introduction

Biomimetic dentin remineralization is an advanced regenerative approach in dentistry that aims to repair damaged teeth by mimicking nature’s biomineralization process. Instead of simply patching cavities, it restores the structural, mechanical, and functional integrity of demineralized dentin [1,2].

In dentin biomimetic remineralization, chemical irrigants act as critical pretreatment conditioning agents. Rather than simply disinfecting, their primary role is to prepare the collagen scaffold, expose endogenous growth factors, and control host enzymes, enabling amorphous calcium phosphate precursor minerals to infiltrate and rebuild tooth structure [3,4]. Successful biomimetic remineralization depends on a balanced irrigant protocol that sufficiently demineralizes the collagen matrix while preserving its structural and biochemical integrity, enabling effective intrafibrillar mineral reconstruction.

Several irrigant solutions can be used in root canal treatment, including ethylenediamine tetraacetic acid (EDTA), citric acid, sodium hypochlorite (NaClO), and chlorhexidine (CHX) [5]. These solutions are germicidal, fungicidal, nontoxic, nonirritating, and stable in solution; do not interfere with tissue repair; have a prolonged antimicrobial effect; and are relatively inexpensive [6]. Some irrigating solutions can alter the organic and inorganic components of dentin [7], leading to decreased bond strength and tissue hardness and changes in surface roughness [8]. The chemical weakening of the root canal could significantly compromise its long-term prognosis [9], leading to sudden root canal fractures under critical loads or in the absence of intracanal posts.

Endodontic irrigants play a key role in dentin biomimetic remineralization by modulating the structural integrity of the collagen scaffold, the degree of demineralization, and the bioavailability of signaling molecules essential for mineral nucleation. The four major clinical irrigants—EDTA, NaClO, citric acid and CHX—may each perform a specific, distinct function in this regenerative sequence. The above-cited solutions are used in subsequent irrigation protocols [10,11]. Recent clinical recommendations report multiple benefits of using an association of irrigants for endodontic healing outcomes, particularly when considering large, infected lesions, the presence of varying amounts of endodontic smear layer covering the dentinal tubules and microbiological niches residing in the deep portions of instrumented/packed root canals [12]. However, using multiple irrigants in root canal treatment can lead to cross-reactions among irrigants, such as deactivation and loss of activity, formation of insoluble or toxic byproducts in the root canal, effervescence, and increased risks of apical extrusions and subcutaneous emphysema [13]. In addition, multi-step protocols could be time-consuming and less acceptable to patients.

The addition of surface modifiers (e.g., surfactants) that act as detergents, emulsifiers, wetting agents, foaming agents, or dispersants may reduce the surface tension of irrigants. The increased surface tension may be useful for longer activity on root canal dentin and may potentially improve irrigant activity, antibacterial effectiveness and clinical performance [14]. More recently, an irrigant formulation combining citric acid, surfactants, and other active components has shown antibiofilm and smear layer removal capabilities, confirming the value of surfactant addition to citric acid-based irrigants [15].

Ethylenediaminetetraacetic acid (EDTA), particularly at low concentrations [16], is considered the most favorable agent, as it selectively chelates the inorganic phase, removes the smear layer [8], and exposes a collagen-rich matrix while preserving its ultrastructure. This environment may support subsequent intrafibrillar remineralization and enhance the release of dentin-derived growth factors such as TGF-β1 and BMPs, which are key mediators of pulp-dentin regeneration [17]. At the same time, EDTA has been shown to minimally affect dentin microhardness and surface roughness even at relatively high concentrations, supporting its use as a biocompatible conditioning agent in regenerative protocols [18].

In contrast, sodium hypochlorite (NaClO), although indispensable for its antimicrobial and tissue-dissolving properties [19], can be detrimental by degrading the organic matrix of dentin [20,21], particularly collagen, thereby compromising the scaffold required for biomimetic mineral deposition. However, its impact on dentin is well documented to be concentration- and time-dependent: NaClO degrades the organic matrix of dentin by oxidizing and hydrolyzing collagen, thereby compromising the scaffold required for biomimetic mineral deposition [22]. Studies using infrared spectroscopy and nanoindentation have confirmed that NaClO treatment significantly reduces dentin collagen content and mechanical properties, including hardness and elastic modulus, effects that are exacerbated at higher concentrations and longer exposure times [8,9].

Chlorhexidine (CHX) is the most commonly used antiseptic product [23]. Most commonly used as a 2% gluconate solution, CHX is the irrigant of choice when NaClO is contraindicated, particularly in cases of hypersensitivity or in regenerative endodontic procedures where preservation of the organic matrix is paramount [24]. Due to its non-toxicity and lack of adverse effects on microhardness and dentin roughness, it was suggested as a suitable irrigant in endodontics [18]. Moreover, it may indirectly contribute to remineralization by inhibiting matrix metalloproteinases (MMPs) and cathepsins (host-derived collagenolytic enzymes responsible for the progressive degradation of the hybrid layer), thereby stabilizing the exposed collagen fibrils, preserving the hybrid layer over time network and potentially extending the longevity of dentin-adhesive interfaces [4,25].

Citric acid, while effective in smear layer removal and dentin demineralization [26], is more aggressive than EDTA: it protonates the phosphate ions of hydroxyapatite, generating HPO_4_^2−^ and H_2_PO_4_^−^ species and inducing substantial mineral loss even at short exposure times [27]. While this demineralizing capacity has been exploited to enhance the release of dentin-derived growth factors in regenerative endodontic procedures [28], excessive demineralization may compromise collagen integrity and reduce the treated substrate's remineralization potential. Furthermore, citrate ions released upon CA–apatite interaction have been shown to adsorb strongly onto apatite nanocrystal surfaces, inhibiting their further growth and potentially hindering the maturation of newly nucleated calcium phosphate phases [29]. These contrasting effects make CA a particularly interesting irrigant to investigate in the context of long-term biomimetic remineralization.

Despite the extensive characterization of these irrigants and their role in promoting adhesion between dentin and the root canal sealer, which ensures the functionality of obturating cones used to fill the root canal [30], previous studies have predominantly focused on short-term mechanical outcomes such as microhardness and flexural strength [8,9], with less attention given to molecular-level spectroscopic assessment of irrigant effects on dentin composition [26]. As a consequence, a systematic, comparative evaluation of the long-term effects of multiple clinical irrigants on dentin biomineralization potential, monitored by infrared spectroscopy, is still lacking. Previous spectroscopic investigations [27,31] were limited to chelating agents (EDTA, citric acid) and shorter ageing periods. As an additional novelty, this study analyses dentin slices obtained from a healthy extracted tooth that was endodontically instrumented in vitro prior to sectioning, to simulate the dentine surface of an instrumented root canal. Moreover, while single-step irrigant formulations combining citric acid and surfactants have recently attracted interest for their simultaneous antibiofilm and smear layer removal capabilities [15], their physicochemical effects on dentin and their influence on long-term biomineralization potential have not been investigated. The present study was therefore designed to fill this gap.

The use of IR spectroscopy in the Attenuated Total Reflection (ATR) mode enabled non-destructive monitoring of both processes and assessment of physicochemical variations in the collagen network and the calcium phosphate matrix. Actually, the IR spectrum reflects contributions from both collagen and apatite phases. The intensities of IR absorption bands provide quantitative information about sample composition, depending on the nature of chemical bonds, their strength, and the environment [26,27,32].

## 2. Materials and Methods

### 2.1. Root Dentin Disk Preparation and Treatment

A caries-free second premolar with a single root canal, extracted for periodontal reasons, was used for the present investigation. Unlike a previous study in which coronal dentinal portions were obtained from extracted third molars [27], the tooth underwent conventional root canal treatment. This tooth was instrumented with a single-file NiTi instrumentation protocol. Briefly, the working length was established using a stainless steel K file #10. Then, a Hyflex EDM single file 25/08 (Hyflex EDM, Coltene, Altstaetten, Switzerland) was inserted into the canal using a progressive movement to reach the working length. After that, the tooth was sectioned using a low-speed water-cooled diamond saw (Micromet; Remet, Bologna, Italy) under constant water cooling to prevent heat-induced damage to the root dentin structure.

A total of six dentine discs (1 mm width × 8 mm length, referred to as cross-sectional cuts) were obtained.

Each dentine disc was immersed in 5 mL of irrigating solution for 10 min and then stored in HBSS at 37 °C for 3 months. The medium was refreshed every 2 weeks [27,32].

The solutions from Coltene (Altstaetten, Switzerland) were the following:

Canal Pro CHX 2%;
Citric Acid 10%;
NaClO 3%;
Experimental irrigant (an aqueous 7% citric acid solution containing two different surfactants as additional active ingredients);
EDTA 17%.

All tested solutions were commercial irrigants, except for the experimental solution, which was designed for single-step use. No information on its biochemical effects on root canal dentin is reported in the literature.

Sterile water was used as a control. A limitation of the present in vitro study is the use of dentin slices obtained from a single extracted tooth. However, this experimental design was chosen to minimize inter-tooth variability in dentin composition and structure, allowing a more controlled comparison of the trends induced by the different irrigants. Further studies are needed to confirm these observations in broader experimental models.

### 2.2. IR Spectroscopy

Dentin slices were analyzed by IR spectroscopy at each step of the study. Dried irrigant solutions were analyzed as a reference for comparison.

IR spectra were recorded in triplicate on a Bruker Alpha Fourier Transform FTIR spectrometer, equipped with a Platinum Attenuated Total Reflectance (ATR) single reflection diamond module (penetration depth 2 µm) and a Deuterated Lanthanum α-Alanine doped TriGlycine Sulfate (DLaTGS) detector; the spectral resolution was 4 cm^−1^; each spectrum was the average of 64 scans. It should be noted that the penetration depth of the ATR accessory (~2 µm) means that all IR spectra reported in this study are representative exclusively of the outermost surface layer of each dentin disc. Physicochemical changes occurring at greater depths (i.e., modifications to the bulk collagen architecture and bulk mineral phase) are not captured by this technique. All results and interpretations must therefore be understood to pertain only to the dentin surface layer.

According to previous studies [31,33], the calcium phosphate (CaP)/collagen ratio and its carbonate content were evaluated through the A_CaP_/A_Amide I_ and A_870_/A_CaP_ absorbance ratios, respectively, where A_CaP_, A_Amide I_ and A_870_ were the absorbances (measured as peak heights) of the ν_3_ PO_4_^3−^ band (around 1000 cm^−1^), Amide I of collagen (at about 1640 cm^−1^) and ν_2_ CO_3_^2−^ band at about 870 cm^−1^, respectively.

These data were analyzed with R statistical software (version 3.5.3; GNU GPL license). The data have a non-Gaussian distribution, so a nonparametric Kruskal–Wallis test was used to assess statistical significance. A Dunn–Bonferroni post-hoc analysis was performed for any dependent variable for which the Kruskal–Wallis test was significant. All evaluation tests were two-tailed, with an alpha level of 0.05. It should be noted that the Kruskal–Wallis test operates on ranked data and does not assume or use sample means; its results are therefore entirely independent of the mean ± SD values reported in this study. Mean ± SD values are reported for descriptive purposes only to aid the reader in appreciating the magnitude and variability of the spectroscopic measurements and must not be interpreted as the basis for, or as consistent with the assumptions of, the rank-based statistical comparisons. Null results (*p* > 0.05) must not be interpreted as evidence of equivalence between treatments, as the study was not statistically powered to detect differences across a biologically representative sample. In fact, it must be noted that the triplicate IR measurements performed on each dentin disc represent technical replicates (repeated measurements of the same biological specimen) rather than biological replicates from independent subjects. As a consequence, the statistical comparisons between irrigant groups reported in this study assess the consistency of the spectroscopic signal across the disc surface within this single biological unit, but cannot capture biological variability in dentin composition, degree of mineralization, or collagen cross-link density.

## 3. Results

### 3.1. CHX

**Figure 1:**
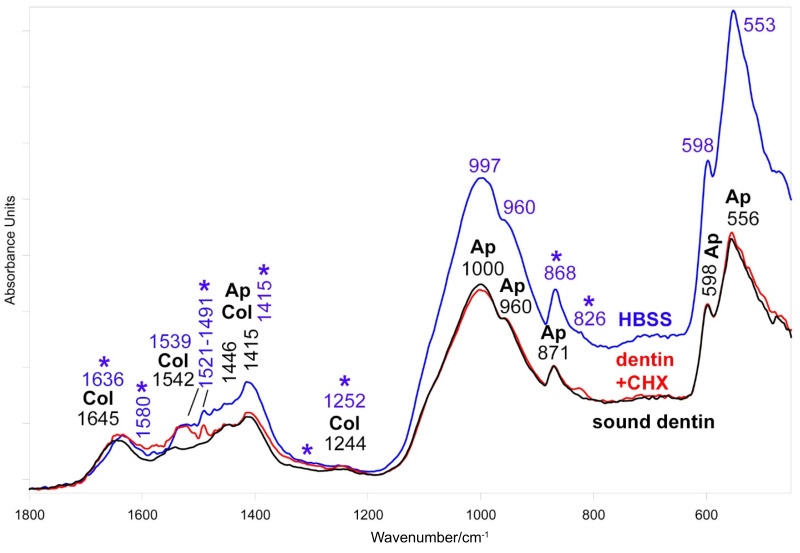
Average IR spectra recorded on the dentin sample before (sound dentin, black line) and after treatment with the CHX solution and washing (red line), and the following ageing in HBSS for three months (blue line). The bands assignable to collagen (Col) and apatite (Ap) are indicated together with those ascribable to CHX (* see Supplementary Material for details).

Figure S1, Supplementary Material shows the IR spectra recorded from the dentin sample before and after treatment with the CHX solution and washing. The spectrum of the dried solution, which displays vibrational features ascribable to chlorhexidine and gluconate ion [34], is reported for comparison to investigate the possible absorption of the irrigant by dentin. Figure 1 also shows the spectrum recorded after three months of ageing in HBSS.

In the spectrum of sound dentin (Figure S1, Supplementary Material and Figure 1), the bands assignable to collagen (in particular Amide I, II, and III bands at 1645, 1542, and 1244 cm^−1^, respectively [35]) have been assigned together with those of B-type carbonated apatite at 1446–1415 cm^−1^ (ν_3_ CO_3_^2−^ antisymmetric stretching), 1000 cm^−1^ (ν_3_ PO_4_^3−^ antisymmetric stretching), 960 cm^−1^ (ν_1_ PO_4_^3−^ symmetric stretching), 871 cm^−1^ (ν_2_ CO_3_^2−^ out-of-plane bending), and 598–556 cm^−1^ (ν_4_ PO_4_^3−^ out-of-plane bending) [36].

Upon irrigant treatment, CHX-attributed bands were detected superimposed on dentin bands, despite subsequent washing.

Interestingly, the CHX bands were also observed after three months of ageing in HBSS (Figure 1).

**Figure 2:**
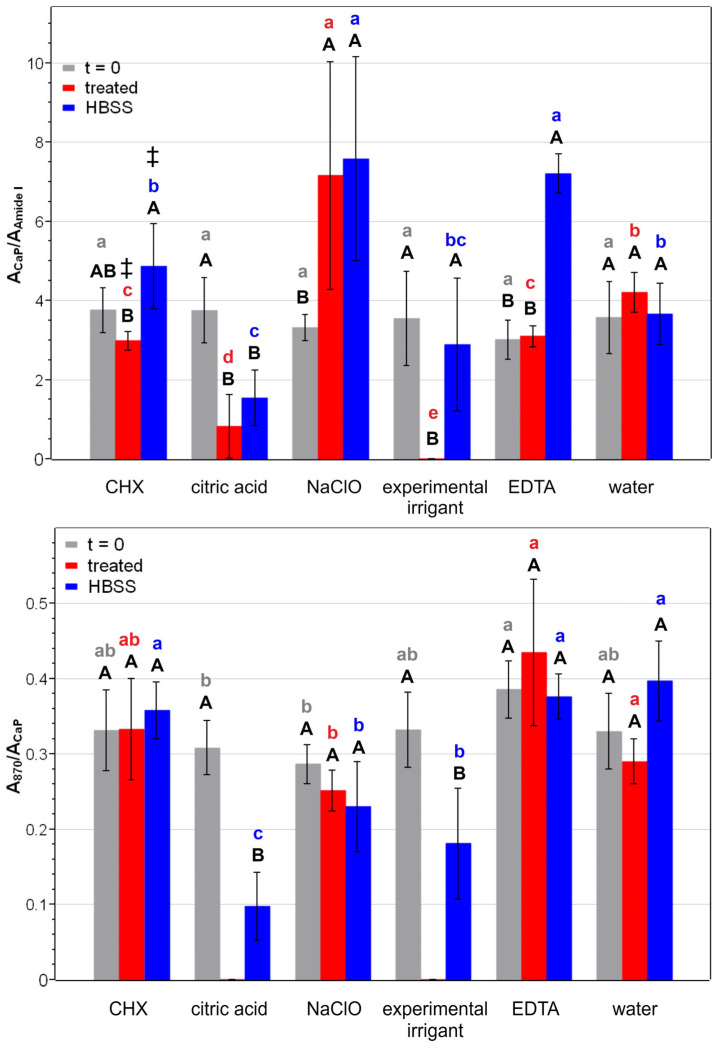
A_CaP_/A_Amide I_ and A_870_/A_CaP_ absorbance ratios (average ± standard deviation, reported for descriptive purposes only) as obtained from the IR spectra recorded on the dentin samples before and after treatment with the different irrigant solutions and washing, and the following ageing in HBSS for three months. Statistical comparisons were performed using the nonparametric Kruskal–Wallis test, followed by Dunn’s post-hoc test with Bonferroni correction for multiple comparisons, applied separately to two pre-specified families of comparisons: within-treatment comparisons across the three time points (t = 0, immediately after irrigant treatment and washing and after 3-month ageing in HBSS) for each irrigant independently (indicated by capital letters) and between-treatment comparisons at the same time point across all six treatment groups (indicated by lowercase letters). Different capital letters indicate statistically significant differences (*p* < 0.05) between values at different time points within each treatment; different lowercase letters indicate statistically significant differences (*p* < 0.05) between values at the same time point across treatments. ^‡^ Values underestimated due to spectral overlap between CHX absorption bands and the collagen Amide I band; direct quantitative comparison with other treatments is not valid.

Due to the contribution of the CHX bands in the spectral ranges where Amide I and Amide II bands fall, the A_CaP_/A_Amide I_ and A_CaP_/A_Amide II_ absorbance ratios, previously identified as spectral markers of the mineralization degree [27,33], did not appear reliable. CHX displays absorption bands at approximately 1580 and 1490 cm^−1^ (aromatic C=C and C–N stretching modes of the bisbiguanide group), which directly overlap with the Amide I (1645 cm^−1^) and Amide II (1542 cm^−1^) bands of collagen, causing the denominator of the A_CaP_/A_Amide I_ ratio to be artificially overestimated [37]. However, to compare the effect of the CHX irrigant with the other treatments, we still decided to calculate the A_CaP_/A_Amide I_ ratio (as reported in Figure 2), aware that this ratio underestimates the real values. The A_870_/A_CaP_ absorbance ratio is identified as a marker of the relative carbonate content in the apatite phase [31] and was considered reliable for all samples since the contribution of CHX to the 870 cm^−1^ band of carbonate should be small (see the relative intensity of this band in Figure S1, Supplementary Material). The values of this ratio are also reported in Figure 2.

Following CHX treatment and washing (Figure 1), the apatite bands did not change significantly in relative intensity compared with collagen; the A_CaP_/A_Amide I_ was not significantly different from that of sound dentin (*p* > 0.05, Figure 2). This trend suggests a substantial constancy in the contents of the organic and inorganic phases under the assumptions reported above.

Concerning the dentin sample aged in HBSS for three months following the irrigant treatment (Figure 1), it may be observed that the apatite bands showed increased relative intensity compared with collagen. Correspondingly, the A_CaP_/A_Amide I_ significantly increased (*p* < 0.05, Figure 2) compared to CHX-treated dentin. Taking into account that this ratio represents an underestimation of the real value, it may be affirmed that these trends suggest calcium phosphate deposition; the ν_3_ phosphate antisymmetric stretching band underwent a wavenumber shift (from 1000 to 997 cm^−1^) and broadening (full width at half maximum, FWHM, increased from 148 to 159 cm^−1^). This downward wavenumber shift of the ν_3_ PO_4_^3−^ band and the increase in its FWHM are consistent with a decrease in the crystallinity and an increase in the carbonate content of the newly deposited apatite phase, since carbonate substitution in the apatite lattice (B-type substitution, where CO_3_^2−^ replaces PO_4_^3−^) is known to shift the ν_3_ phosphate band to lower wavenumbers and broaden it [38]. This is consistent with the deposition of a poorly crystalline, carbonate-substituted apatite during the early stages of biomimetic remineralization, as previously reported [27,31]. No significant changes were observed in the A_870_/A_CaP_ ratio (*p* > 0.05, Figure 2).

### 3.2. Citric Acid

**Figure 3:**
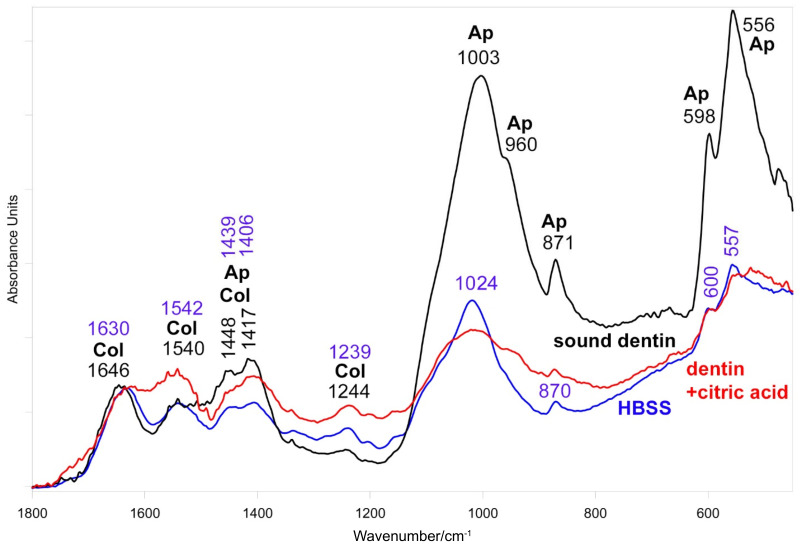
Average IR spectra recorded on the dentin sample before (sound dentin, black line) and after treatment with the citric acid solution and washing (red line, see Supplementary Material for detailed band assignments) and the following ageing in HBSS for three months (blue line). The bands assignable to collagen (Col) and apatite (Ap) are indicated.

Figure S2, Supplementary Material, shows the IR spectra recorded on the dentin sample before and after treatment with the citric acid solution and washing. The spectrum of the dried solution, which displays the vibrational bands typical of citric acid [34], is reported for comparison. Figure 3 also shows the spectrum recorded after three months of ageing in HBSS.

Upon the treatment with the irrigant, the bands of apatite significantly decreased in intensity without disappearing; the ν_3_ phosphate antisymmetric stretching shifted from 1003 to 1021 cm^−1^ (Figure S2, Supplementary Material) and broadened towards the higher wavenumber values, suggesting the formation of HPO_4_^2−^ and H_2_PO_4_^−^ upon protonation of the apatite PO_4_^3−^ ion by citric acid; spectral features assignable to these ions were detected, as detailed in Figure S3, Supplementary Material. In sound dentin apatite, the ν_3_ PO_4_^3−^ antisymmetric stretching appears around 1000–1005 cm^−1^. Upon protonation by citric acid, PO_4_^3−^ is converted to HPO_4_^2−^ and H_2_PO_4_^−^, whose ν_3_ stretching modes appear at higher wavenumbers (approximately 1050–1150 cm^−1^), causing the overall band to shift and broaden towards higher wavenumbers. This is a well-established spectroscopic marker of acid attack on apatite [39,40] and is consistent with the proton-transfer reaction between citric acid and the apatite surface. Dentin demineralization was also indicated by a significant decrease (*p* < 0.05) in the A_CaP_/A_Amide I_ absorbance ratio (Figure 2). The A_870_/A_CaP_ absorbance ratio, identified as a marker of the relative content of carbonate in the apatite phase [31], was not calculated due to the contribution of the HPO_4_^2−^ and H_2_PO_4_^−^ ions to the intensity of the 870 cm^−1^ band (Figure S3, Supplementary Material); from a qualitative point of view, this band appeared to weaken upon treatment. Spectral features assignable to the antisymmetric and symmetric COO^−^ stretching modes of citrate were detected by the strengthening around 1540 and 1400 cm^−1^, respectively (Figure S2, Supplementary Material), suggesting apatite calcium chelation. The COOH stretching band of citric acid (Figure S2, Supplementary Material) was still detectable at about 1720 cm^−1^.

After ageing in HBSS for three months (Figure 3), the citrate/citric acid bands disappeared, and the HPO_4_^2−^ and H_2_PO_4_^−^ vibrational modes became less prominent but remained detectable. The disappearance of the citrate COO^−^ bands upon ageing may be explained by a gradual desorption of citrate ions from the apatite surface into the HBSS medium during the three-month immersion, while the persistence of HPO_4_^2−^/H_2_PO_4_^−^ spectral features even after three months confirms that citrate prevented complete conversion of these precursor species to mature carbonated apatite, as previously observed [29,41]. The ν_3_ phosphate antisymmetric stretching narrowed (its FWHM decreased from 164 to 112 cm^−1^). The maximum of the latter band remained upshifted with respect to sound dentin (1024 cm^−1^ versus 1003 cm^−1^). The A_CaP_/A_Amide I_ absorbance ratio (Figure 2) increased but not significantly and did not reach the value observed in sound dentin. The A_870_/A_CaP_ absorbance ratio was significantly lower (*p* < 0.05) than in sound dentin (0.10 ± 0.05 *versus* 0.31 ± 0.04), suggesting a decrease in the carbonate content in the nucleated phase. The Amide I band of collagen shifted to lower wavenumbers relative to sound dentin, but its FWHM did not change significantly; the other collagen bands also shifted.

### 3.3. Sodium Hypochlorite

**Figure 4:**
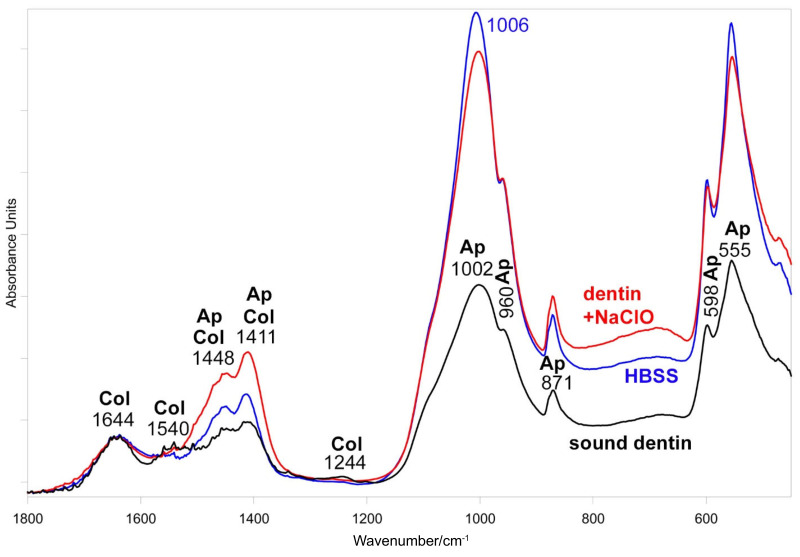
Average IR spectra recorded on the dentin sample before (sound dentin, black line) and after treatment with the NaClO solution and washing (red line), and the following ageing in HBSS for three months (blue line). The bands assignable to collagen (Col) and apatite (Ap) are indicated.

Figure 4 shows the IR spectra of the dentin sample recorded before and after treatment with the sodium hypochlorite solution and washing and after subsequent ageing in HBSS for three months.

Upon treatment with the irrigant solution, the relative intensity of the apatite bands increased due to solubilization of the collagen phase. Correspondingly, the A_CaP_/A_Amide I_ absorbance ratio increased significantly (*p* < 0.05). The crystallinity of the apatite phase increased, as evidenced by a decrease in the FWHM of the ν3 antisymmetric phosphate band from 138 to 118 cm^−1^. This apparent paradox (an irrigant that damages dentin yet increases apatite crystallinity) can be explained by considering that NaClO selectively removes the organic collagen matrix that normally encapsulates and constrains the apatite crystallites. As the organic encapsulation is removed, the remaining apatite crystallites are partially freed from their structural constraints and may undergo limited reorganization [42]. The carbonate content of the remaining apatite phase did not change: the A_870_/A_CaP_ absorbance ratio was the same as in sound dentin. The Amide I band of collagen did not exhibit a significant wavenumber shift, but showed a significant increase in FWHM (from 61 to 71 cm^−1^), indicating increased structural heterogeneity of the collagen phase, consistent with partial conformational changes in the collagen backbone induced by NaClO treatment. It must be noted that the collagen bands remained detectable, suggesting that dentin deproteinization was incomplete within the first 2 microns of the sample thickness.

Upon ageing in HBSS (Figure 4), the relative intensity of the apatite bands increased slightly and, correspondingly, the A_CaP_/A_Amide I_ absorbance ratio increased (Figure 2). However, statistical analysis revealed that this change was not significant (*p* > 0.05). The trend in the A_870_/A_CaP_ absorbance ratio indicated that the carbonate content of the apatite phase did not change significantly (*p* > 0.05). The FWHM of the ν_3_ antisymmetric phosphate band and Amide I bands decreased (from 114 to 108 cm^−1^ and from 71 to 65 cm^−1^, respectively).

### 3.4. Experimental Irrigant

Figure S4, Supplementary Material, shows the IR spectrum of the dried experimental irrigant solution. Bands assignable to citric acid were detected together with those of an organic aromatic component, tentatively identified as 2-phenoxyethanol by comparison with its spectrum. Moreover, Triton surfactants, reported as additives in irrigants [43], exhibit IR bands at similar wavenumbers [34]. It must be noted that this identification is tentative and based solely on spectral comparison with a reference spectrum; it has not been confirmed by independent analytical methods such as mass spectrometry or nuclear magnetic resonance spectroscopy. The presence of Triton-family surfactants cannot be excluded. Given this uncertainty, all mechanistic interpretations involving the surfactant must be regarded as speculative.

Figure S5, Supplementary Material, shows the IR spectra recorded on the dentin sample before and after treatment with the experimental irrigant and washing. The spectrum of a demineralized dentin slice (i.e., collagen) is reported for comparison.

No bands assignable to the apatite phase were detected in the spectrum of the dentin slice treated with the irrigant and then washed (i.e., the A_CaP_/A_Amide I_ absorbance ratio became equal to zero, Figure 2); this result suggests that the treatment solubilized the inorganic phase of dentin in the first 2 microns from the surface, at least (i.e., in the sampling depth of the ATR accessory). No bands assignable to protonated citric acid (in particular, the COOH stretching mode at about 1700 cm^−1^) were detected. Conversely, as observed for the treatment with commercial citric acid solution, bands assignable to citrate were observed, and may be interpreted, as above, as a sign of calcium chelation. Some bands assignable to the surfactant were identified.

**Figure 5:**
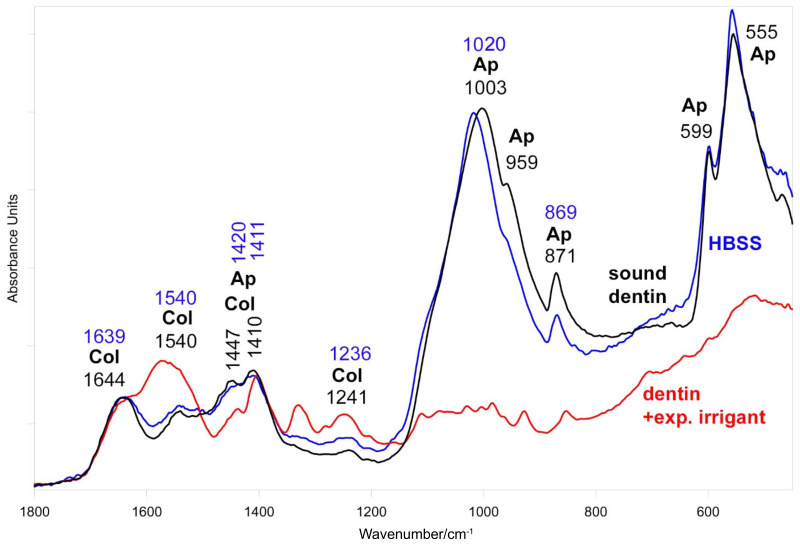
Average IR spectra recorded on the dentin sample before (sound dentin, black line) and after treatment with the experimental irrigant and washing (red line, see Supplementary Material for detailed band assignments), and the following ageing in HBSS for three months (blue line). The bands assignable to collagen (Col) and apatite (Ap) are indicated.

After three months of ageing in HBSS (Figure 5), apatite bands appeared, and the spectral profile was similar to that of sound dentin. Accordingly, the A_CaP_/A_Amide I_ absorbance ratio increased significantly compared with the previous step of the test (i.e., irrigant treatment and washing). It attained a value not significantly different (*p* > 0.05) from that of sound dentin (Figure 2). Some shifts in band wavenumbers were observed (Figure 5). In particular, the ν_3_ phosphate antisymmetric stretching appeared upshifted with respect to sound dentin (1020 cm^−1^ *versus* 1003 cm^−1^), as previously observed for the citric acid treatment. The A_870_/A_CaP_ absorbance ratio was significantly lower than in sound dentin (*p* < 0.05, Figure 2), as previously observed for the citric acid treatment.

### 3.5. EDTA

**Figure 6:**
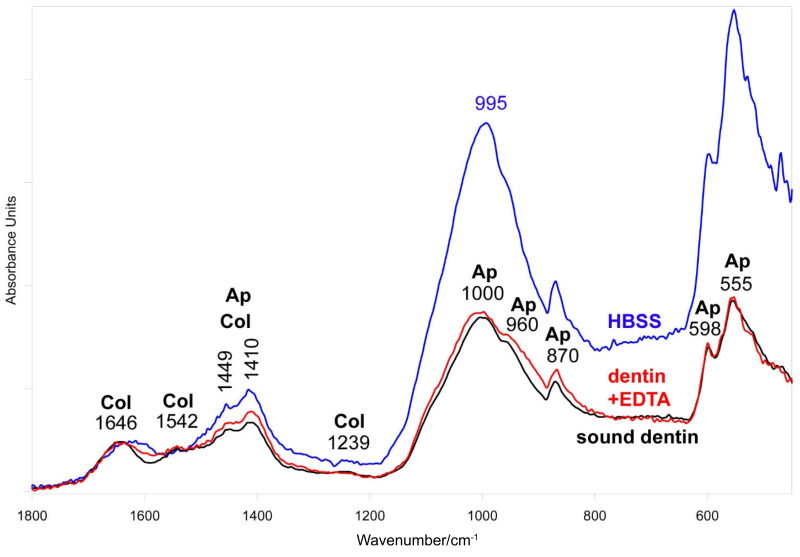
Average IR spectra recorded on the dentin sample before (sound dentin, black line) and after treatment with the EDTA solution and washing (red line), and the following ageing in HBSS for three months (blue line). The bands assignable to collagen (Col) and apatite (Ap) are indicated.

Figure 6 shows the IR spectra recorded from the dentin sample before and after treatment with the EDTA solution, washing and subsequent ageing in HBSS for three months.

Upon the EDTA treatment and washing, only subtle changes were observed in the spectral profile, and no bands ascribable to EDTA adsorption were detected, in agreement with previous studies [31,33]; only a slight broadening of the Amide I and ν_3_ antisymmetric phosphate stretching modes was observed. No significant variations in the A_CaP_/A_Amide I_ and A_870_/A_CaP_ absorbance ratios occurred (Figure 2).

Upon ageing in HBSS for three months, a significant strengthening of the apatite bands was observed (Figure 6), together with a corresponding increase in the A_CaP_/A_Amide I_ absorbance ratio (Figure 2). Amide I shifted to lower wavenumbers (Figure 6).

### 3.6. Water

Figure S6, Supplementary Material, shows the IR spectra recorded on the dentin control sample (treatment with water) before and after ageing in HBSS for three months.

Upon ageing, no significant changes in the spectral profile were observed; correspondingly, the A_CaP_/A_Amide I_ and A_870_/A_CaP_ absorbance ratios did not vary significantly (Figure 2).

## 4. Discussion

Vibrational spectroscopy allowed for greater insights into the effects of irrigants on dentin at both short-term (i.e., immediately after the irrigation procedures) and long-term (after three months of ageing in HBSS).

The present study demonstrated that the short-term behavior of dentin under irrigant treatment influenced its long-term behavior. It should be noted that the irrigants investigated in this study exhibit fundamentally different chemical modes of action. Citric acid promotes dentin demineralization through proton donation and calcium chelation, EDTA acts predominantly by calcium complexation at near-neutral pH, NaClO exerts oxidative degradation of the organic matrix under strongly alkaline conditions, whereas CHX interacts mainly through adsorption of its cationic molecules onto dentin. Therefore, differences in dentin behavior cannot be attributed to a single physicochemical parameter such as pH or degree of dissociation but rather to the distinct reaction mechanisms of each irrigant.

### 4.1. Irrigant Treatment and Washing: Effects of the Irrigants on Dentin

The irrigants that were found to alter dentin to the lowest extents were CHX and EDTA; after the treatment with these solutions and following washing, no significant changes in the relative intensities of the collagen and apatite bands were observed (Figure 1, Figure 6 and Figure S1, Supplementary Material), suggesting that their relative contents remained unchanged; these qualitative findings were confirmed more quantitatively by the trend of the A_CaP_/A_Amide I_ absorbance ratio (Figure 2). Concerning CHX, this result confirms that CHX does not significantly solubilize the organic and inorganic phases of dentin [24]. Regarding EDTA, the constancy of apatite and collagen contents may be attributed to the relatively short treatment duration. Analogous results were obtained after treatment with EDTA of similar and lower concentrations for 1’ [27]. At the same time, complete demineralization down to a thickness of 2 microns was observed when the treatment lasted 2 h [31,33]. This time-dependence of EDTA demineralization is explained by the chelation kinetics of EDTA with calcium ions in the apatite lattice: at short exposure times, EDTA preferentially removes calcium from the outermost surface, leaving a partially demineralized layer; at longer exposure times, the chelation front progressively advances into the specimen. The short treatment duration used in the present study (10 min) is clinically representative of standard endodontic protocols, where final EDTA irrigation is typically applied for 1–3 min [10] and is therefore appropriate for evaluating clinically relevant conditioning effects.

The vibrational data, particularly the trend in the A_CaP_/A_Amide I_ absorbance ratio, showed that the citric acid-based irrigants induced the highest dentin demineralization (Figure 2), consistent with a previous study [27]. A key finding of this study is that the two citric acid-based irrigants, despite sharing the same active demineralizing agent (citric acid), produced markedly different short-term effects on dentin composition, attributable to the presence of a surfactant in the experimental formulation. This comparison is particularly informative because it allows the specific contribution of the surfactant to be isolated and evaluated independently of the acid chemistry. The commercial (i.e., pure) citric acid solution, despite its higher concentration, was unable to completely demineralize dentin in the first 2 microns of depth. Correspondingly, the A_CaP_/A_Amide I_ absorbance ratio decreased significantly but did not reach zero (Figure 2), and the formation of an H_2_PO_4_^−^/HPO_4_^2−^-containing apatite was spectroscopically revealed (Figure 3, Figures S2 and S3, Supplementary Material). On the contrary, the experimental irrigant, although less concentrated (7% *versus* 10%), successfully demineralized dentin within the first 2 microns of depth. As shown in Figure 5 and Figure S5, Supplementary Material, the apatite bands became undetectable upon treatment with the experimental irrigant and following washing; correspondingly, the A_CaP_/A_Amide I_ absorbance ratio equaled zero (Figure 2). This different behavior may be attributed to the presence of a surfactant in the experimental irrigant formulation. Evidently, this ingredient, which was found adsorbed on the dentin surface (Figure S5, Supplementary Material), facilitated more efficient permeation of citric acid into the sample depth. This different behavior is consistent with the presence of a surface-active component in the experimental irrigant formulation, which, regardless of its precise chemical identity, appears spectroscopically to have been adsorbed onto the dentin surface (Figure S5, Supplementary Materials). Surface-active agents are known to reduce surface tension and enhance the permeation of active ingredients into dentinal tubules [15,44,45,46], and this general mechanism provides a plausible explanation for the observed enhanced demineralization. However, since the identity of the surfactant could not be confirmed beyond tentative spectroscopic matching, this interpretation must be regarded as a working hypothesis that requires verification through studies using formulations with known surfactant compositions. An analogous increase in tissue-dissolving effectiveness upon the addition of surfactants was observed in previous studies of NaClO-based solutions [44,45,46]. On the other hand, a more recent study showed that Triton can provide dual benefits of antibiofilm and smear layer removal capabilities simultaneously, indicating a simplified and effective strategy for application in root canal treatment [15].

CHX and citric acid were found to be adsorbed by the dentin surface (Figures S1, S2, and S5, Supplementary Material). CHX bands were still detected after three months of ageing in HBSS (Figure 1). This result was not unexpected, since it is well known that dentin adsorbs CHX [47]. Concerning citric acid, this ingredient was adsorbed as such (i.e., detection of the COOH stretching band of protonated citric acid at about 1720 cm^−1^) when dentin was treated with the pure citric acid solution (i.e., the most concentrated one). On the contrary, the 1720 cm^−1^ band was not detected upon treatment with the less concentrated experimental citric acid-based irrigant. In both cases, the citrate bands were detected immediately after the irrigant treatment and washing, while they disappeared after ageing for three months in HBSS. The formation of citrate may be explained by considering that citric acid reacts with the dentin apatite by protonating the phosphate ion, thereby losing a proton; upon loss of a proton, the citrate ion forms. Actually, upon treatment with the pure citric acid solution, i.e., the less-penetrating irrigant, an H_2_PO_4_^−^/HPO_4_^2−^-containing apatite was observed on the surface (i.e., in the first 2 microns of depth) of the sample (Figure 3, Figures S2 and S3, Supplementary Material).

The detection of citrate bands suggests apatite calcium chelation [48]; citrate ions have also been reported to be adsorbed on bone apatite through their COO^−^ groups [29]. This interpretation is particularly true for the sample treated with the pure citric acid solution, whose spectrum still showed apatite bands (Figure 3 and Figure S2, Supplementary Material). Citrate ions were also detected on the surface of the sample treated with the experimental irrigant (Figure S5, Supplementary Material), whose spectrum showed no apatite band. In this case, the detection of citrate ions in the absence of apatite may be explained by citrate’s reported ability to bind to collagen [48].

Sodium hypochlorite partially solubilized the organic collagen phase [22]; actually, NaClO was the only irrigant that induced an increase in the A_CaP_/A_Amide I_ absorbance ratio (Figure 1) due to its ability to degrade the dentin organic component by infiltrating into the apatite-encapsulated collagen matrix. The same spectroscopic intensity ratio was used in a previous study to assess the decrease in the surface collagen fraction by 40% within 2 min of NaClO exposure [49]. However, it may be observed that NaClO also affected the inorganic apatite phase of dentin, which increased its crystallinity (decreased FWHM of the ν_3_ phosphate band) due to a possible reorganization of apatite crystallites without changing its carbonate content. For this purpose, controversial data are reported in the literature due to marked differences in the proposed experimental protocols (NaClO concentration, treatment duration, tissue provenance, and physical form of the substrate, i.e., bulk sample or powder…). Past investigations had demonstrated that NaClO exclusively affected the organic phase of dentin without altering the apatite lattice [42,50,51,52]. Regarding its carbonate content, our result agrees with the trend reported by several studies [50,51,52] but differs from the findings of Tartari et al., who observed a decrease [53].

An important methodological consideration common to all results reported in this study is the ~2 µm sampling depth of the ATR accessory. This depth corresponds to the outermost surface layer of the dentin disc, a region that includes the instrumentation smear layer and the immediately underlying peritubular and intertubular dentin. While this surface layer is directly relevant to the initial irrigant–dentin interaction, it represents only a small fraction of the clinically relevant dentin wall thickness. The spectroscopic changes reported in the present study about demineralization, apatite crystallinity changes, collagen band shifts, and remineralization may therefore not accurately reflect the changes occurring at greater depths within the dentin wall. In particular, irrigants with known deep penetration capability, such as NaClO, may have produced collagen degradation and mineral alterations well beyond the 2 µm ATR sampling depth that remained undetected in this study. Conversely, the remineralization observed after ageing in HBSS may have been confined to the outermost surface layer, with deeper dentin regions remaining unaffected. These considerations reinforce the preliminary nature of all findings and underscore the need for complementary depth-sensitive analytical techniques.

### 4.2. Ageing in HBSS

The long-term (i.e., three-month) ageing test in HBSS was performed to monitor the activity of root canal irrigants after their application, simulating the clinical period preceding prosthetic rehabilitation. The hypothesis was that prolonged irrigation could irreversibly alter organic collagen and induce biochemical damage that could lead to sudden weakening (and fracture) of the root.

After ageing in HBSS for three months, the relative intensity of the apatite bands increased to varying degrees depending on the irrigant treatment (Figure 1, Figure 3, Figure 4, Figure 5 and Figure 6). The dentin slices previously treated with CHX and EDTA (i.e., those that underwent the lowest changes upon the treatment) underwent mineralization, attaining apatite/collagen content ratios (i.e., A_CaP_/A_Amide I_ values) on average higher than sound dentin, although the statistical significance of this difference was not formally assessed (Figure 1, Figure 2 and Figure 6). This observation suggests a correlation between the degree of short-term structural perturbation induced by the irrigant and the subsequent remineralization capacity: irrigants that preserve the structural integrity of both the organic and inorganic dentin phases appear to create the most favorable conditions for subsequent apatite nucleation [16,18,21].

Upon these treatments, the carbonate content of the CaP phase remained the same as in sound dentin. It must be stressed that the control dentin sample, i.e., that was treated with water (i.e., no irrigant applied), did not show an analogous remineralization upon ageing in HBSS (Figure 2 and Figure S6, Supplementary Material). This trend reinforced the idea that the irrigant treatment was essential for the subsequent remineralization. Concerning the EDTA treatment, a slight broadening of the Amide I and ν_3_ antisymmetric phosphate stretching modes was observed (Figure 6); evidently, these changes were sufficient to trigger apatite deposition during subsequent ageing in HBSS. In the context of biomimetic remineralization, the increased disorder indicated by the broadening of spectral bands may indicate a partial disruption of the collagen helices, which may expose calcium-binding carboxylate groups (Asp and Glu residues) acting as seeds for subsequent mineral growth [27,31].

Regarding the CHX treatment, the induced remineralization may be explained by the IR-detected adsorption of the irrigant (Figure 1); the adsorbed charged groups of CHX may act as templates for apatite deposition. The persistence of CHX bands in the IR spectra after three months of ageing in HBSS (Figure 1) is significant because it confirms that the A_CaP_/A_Amide I_ ratio is underestimated in CHX-treated samples due to spectral overlap, as discussed in Section 3.1. Moreover, the long-term retention of CHX on the dentin surface suggests that the adsorbed charged groups of CHX may serve as heterogeneous nucleation sites for calcium phosphate deposition, thereby contributing to the significant remineralization observed upon ageing in HBSS. A previous study [54] quantified CHX retention in mineralized, partially demineralized, and totally demineralized dentin discs over periods of up to eight weeks, demonstrating that significant amounts of CHX remained bound to all dentin substrates regardless of the applied dose or incubation time, with the outstanding substantivity attributed to its inhibitory effect on dentinal proteases. This study directly supports our spectroscopic observation of CHX persistence and the interpretation that adsorbed CHX may act as a nucleation template for apatite deposition.

Despite the complete demineralization induced by the experimental irrigant in the first 2 microns of dentin depth, a significant remineralization occurred upon ageing in HBSS for three months, suggesting that the apatite nucleating ability was not lost upon the irrigant treatment. As can be seen in Figure 2, the A_CaP_/A_Amide I_ absorbance ratio typical of sound dentin was recovered upon ageing in HBSS for three months, while the carbonate content of the nucleated apatite phase was lower than that in sound dentin, as revealed by the lower value of the A_870_/A_CaP_ absorbance ratio, in agreement with a previous study [27]. The treatment with pure citric acid was less favorable. Following ageing in HBSS, the A_CaP_/A_Amide I_ absorbance ratio did not increase significantly (Figure 2), suggesting no detectable apatite nucleation. The apatite/collagen ratio typical of sound dentin was not recovered, and the carbonate content of the CaP phase was lower than in sound dentin (Figure 2).

As reported above, the treatment with NaClO partially solubilized the organic phase of dentin; at the same time, the remaining collagen showed spectroscopic evidence of increased structural disorder, as indicated by the broadening of its Amide I band (Figure 4); while this observation is consistent with partial denaturation of collagen induced by NaClO [22], definitive characterization of the secondary structure changes would require second-derivative spectroscopy or curve-fitting analysis of the Amide I band envelope, which were not performed in this study. Evidently, these changes hindered remineralization; in fact, upon subsequent ageing in HBSS, the A_CaP_/A_Amide I_ absorbance ratio did not change significantly, nor did A_870_/A_CaP_ (Figure 2). In a previous paper [31], we studied EDTA-demineralized dentin soaked in HBSS and DPBS in contact with calcium silicate cements. In that context, we considered that collagen conformational integrity was a prerequisite for effective apatite nucleation. This finding may support our interpretation that the conformational rearrangements of collagen induced by NaClO, as evidenced by the broadening of the Amide I band, compromise its nucleating ability during ageing in HBSS.

**Figure 7:**
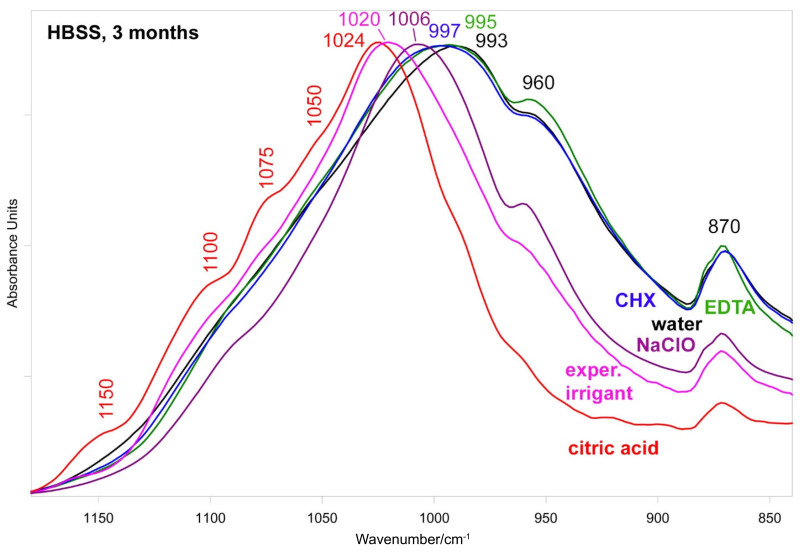
Average IR spectra recorded on the differently treated dentin slices aged in HBSS for three months.

Treatment with different irrigants affected not only the amount of CaP phase formed upon ageing in HBSS (as discussed above) but also its chemical nature. To gain further insight into this aspect, Figure 7 compares the IR spectra of dentin slices treated differently and aged in HBSS for three months.

**Figure 8:**
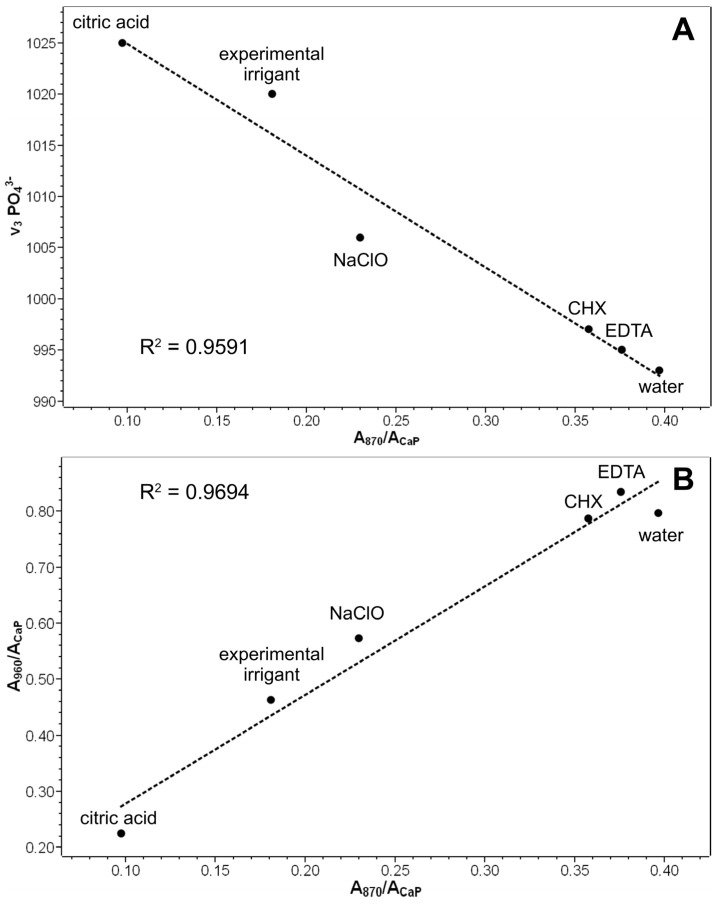
Trend of the ν_3_ phosphate band wavenumber position (**A**) and A_960_/A_CaP_ absorbance ratio (**B**) as a function of the A_870_/A_CaP_ absorbance ratio, as obtained from the IR spectra recorded on the differently treated dentin slices aged in HBSS for three months (reported in Figure 7). Dashed lines represent the linear regression trend lines through the experimental data points; R^2^ values are the coefficients of determination, indicating how well the linear regression fits the data.

The most evident differences concerned the wavenumber position of the ν_3_ phosphate antisymmetric stretching band around 1000 cm^−1^, the relative intensities of the ν_1_ phosphate symmetric stretching band at about 960 cm^−1^, and ν_2_ carbonate out-of-plane bending at 870 cm^−1^. These spectral features appeared to be correlated, as evidenced by the strong linearity of the graphs shown in Figure 8. These graphs show that with increasing carbonate content (i.e., when increasing the A_870_/A_CaP_ absorbance ratio), the ν_3_ phosphate band shifted to lower wavenumbers, the ν_1_ phosphate band strengthened, and, thus, the A_960_/A_CaP_ increased. Similar trends have already been observed in synthetic B-type carbonated apatites with different carbonate contents [38].

These differences suggest that the phases formed on the dentin slices upon ageing in HBSS have different degrees of maturation. In this context, it may be recalled that carbonate naturally substitutes into the apatite lattice over time, and the total carbonate content of the mineralized tissues increases with age/maturation [55]. Consistent with these trends, the absorbance ratios of CHX- and EDTA-treated samples were the closest to sound dentin in Figure 8, indicating that the calcium phosphate phases formed after ageing most closely resembled the composition and maturation state of native dentin. In contrast, the sample treated with pure citric acid showed the greatest deviation from sound dentin, reflecting the persistence of immature phosphate precursor species and a reduced degree of carbonate substitution. These observations further support the main finding of the present study that irrigants preserving the dentin collagen scaffold and promoting balanced mineral nucleation (particularly CHX and EDTA) provide more favorable conditions for long-term biomimetic remineralization than citric acid alone.

Based on the above-discussed spectral features, it may be affirmed that the phases formed on the dentin treated with the citric acid-based irrigants appeared less mature than on the other samples. This fact appears particularly evident for the dentin slice treated with the pure citric acid solution, which, after ageing in HBSS (Figure 7), showed prominent spectral features assignable to H_2_PO^4−^/HPO_4_^2−^ ions in the 1050–1150 cm^−1^ range (see Figure S3, Supplementary Material for assignments). These ions have been identified in the early stages of mineralization, and the CaP phases that contain them are precursors to apatite deposition [39,56,57]. The lower degree of maturation of the phases formed upon treatment with citric acid-based irrigants may be explained by considering that citrate ions, which have been spectroscopically revealed (Figures S2 and S5, Supplementary Material), have been found to restrict the growth of apatite and the transformation of HPO_4_^2^—containing calcium phosphates into hydroxyapatite [40] because of their strong chelation ability with calcium ions [41]. Citrate ions have been reported to bind to the nanocrystal surface, stabilizing apatite nanocrystals by preventing further growth [51]. These aspects may explain why, upon ageing in HBSS, the dentin slice treated with pure citric acid solution did not undergo any significant remineralization and no significant increase in the A_CaP_/A_Amide I_ absorbance ratio was observed (Figure 2); evidently, the apatite crystals revealed on the surface of the sample after the irrigant treatment and washing (Figure 3 and Figure S2, Supplementary Material) became unable to nucleate a CaP phase due to the inhibitory action exerted by the chelated citrate ions. A previous solid-NMR study [29] demonstrated that citrate is strongly bound to the surface of bone apatite nanocrystals and proposed that this binding stabilizes the nanocrystals, preventing the formation of additional phosphate layers. This finding could provide a molecular-level explanation for the inhibition of apatite crystal growth we observed in citric acid-treated dentin after ageing in HBSS. From this point of view, the significant remineralization of the dentin slice treated with the experimental irrigant (which contained citric acid as well) could seem unexplainable; as stressed above, upon the irrigant treatment and washing, this sample underwent a complete demineralization within the first 2 microns of depth (Figure 5 and Figure S5, Supplementary Material), and citrate ions bound to collagen were detected. A significant remineralization occurred upon the following ageing in HBSS. The results of this test may be explained in relation to the findings by Rhee et al. [48]. These authors have reported that citrate bound to collagen, unlike neat collagen [58], has a strong ability to induce apatite formation in vitro when present in appropriate amounts (i.e., in the range 0.3–2 mM). Therefore, citrate might play an important role not only in stabilizing existing apatite nanocrystals but also in crystal nucleation during biomineralization, providing a mechanistic explanation that is independent of the surfactant identity. The possible additional contribution of the adsorbed surfactant to apatite nucleation (suggested by the detection of surfactant bands on the dentin surface after treatment and washing, as shown in Figure S5, Supplementary Material) represents a secondary, speculative hypothesis that cannot be substantiated without knowledge of the surfactant’s precise chemical identity and surface chemistry. Future studies using model formulations with known surfactant compositions, or using the experimental irrigant with and without its surfactant component, would allow the specific contribution of the surfactant to be isolated and quantified.

The absence of any detectable mineral deposition on the water-treated control dentin during three-month ageing in HBSS requires explicit explanation, since HBSS is supersaturated with respect to hydroxyapatite. This apparent paradox is resolved by considering that thermodynamic supersaturation is necessary but not sufficient for spontaneous mineral precipitation: after the critical ionic activity product for homogeneous nucleation, mineral deposition requires heterogeneous nucleation on a template surface that lowers the activation energy barrier [59]. Intact sound dentin, with its smear layer fully preserved following water treatment, does not provide such a template: the smear layer physically occludes access to the underlying collagen and apatite surfaces. This physicochemical barrier was not overcome by simple immersion in HBSS in the absence of any surface-activating treatment. Conversely, all irrigant treatments, even those causing minimal compositional changes such as CHX and EDTA, modified the dentin surface in ways that lowered the effective nucleation barrier by partially disrupting the smear layer, exposing collagen or residual apatite crystallites, or adsorbing nucleation-promoting species onto the surface. The water control result therefore provides important negative evidence confirming that irrigant pretreatment is a necessary and active condition for subsequent biomimetic remineralization in HBSS, rather than a passive permissive condition.

Overall, the results and most relevant trends observed in the present investigation are summarized in Table 1.

The present study focused on the effects of irrigant solutions on dentin and did not evaluate cellular responses. However, it is important to recall possible side effects when irrigants are apically extruded. In particular, NaClO may induce marked cellular damage due to its proteolytic and oxidative activity, while CHX may affect cell membrane integrity and reduce cell viability. NaClO accidents are a real emergency in clinical practice [60]. A recent study showed that concentrations of sodium hypochlorite > 1.5%, chlorhexidine > 2%, citric acid > 10%, and EDTA > 2.5% significantly reduced apical papilla stem cell viability [61]. Recent trends in endodontic irrigations proposed attractive strategies to reduce post-operative pain occurrence related to irrigants and limit apical extrusion events, such as lowering the concentration or modifying the temperature of irrigants or use of irrigant activation strategies (passive irrigation) [62].

## 5. Conclusions

This study demonstrated that the short-term physicochemical impact of endodontic irrigant treatment on root dentin is a critical determinant of its long-term biomineralization potential, as assessed by ATR-FTIR spectroscopy. Among the irrigants tested, CHX and EDTA caused the least structural alteration to the collagen and apatite phases and promoted the most significant remineralization upon three-month ageing in HBSS, with recovery of apatite/collagen ratios exceeding those of sound dentin and formation of a well-crystallized, carbonated apatite phase. NaClO, while clinically indispensable for its antimicrobial and tissue-dissolving properties, partially degraded the collagen scaffold and hindered subsequent apatite nucleation, confirming that its use should be carefully controlled in regenerative endodontic protocols. Commercial citric acid induced marked demineralization but poor remineralization due to the inhibitory effect of citrate ions adsorbed on residual apatite crystals. Conversely, the experimental irrigant promoted significant remineralization primarily attributable to citrate–collagen binding [48]; a possible additional contribution of the adsorbed surfactant component cannot be excluded but remains speculative given the tentative nature of its spectroscopic identification. Clarification of the surfactant identity and its specific role in apatite nucleation represents an important objective for future work. These findings provide molecular-level evidence that irrigant selection profoundly influences dentin biomineralization capacity and suggest that single-step formulations combining citric acid with surfactants represent a promising avenue for future development in regenerative endodontics. These findings must be regarded as preliminary, given the single-tooth design of this study. They provide a controlled and consistent spectroscopic framework linking irrigant-induced short-term dentin modifications to long-term biomineralization potential and generate testable hypotheses for future studies employing multi-donor designs and complementary analytical techniques such as scanning electron microscopy and nanoindentation.

## Figures and Tables

**Table 1:** Summary of the main short-term (immediately after irrigant treatment) and long-term (3-month ageing in HBSS) effects of the tested irrigants on root dentin composition and biomineralization potential, as assessed by ATR-FTIR spectroscopy.

Irrigant	Short-Term Effect	3-Month Ageing in HBSS	Overall Interpretation
CHX	Limited changes in the relative contribution of the mineral and organic phases. CHX bands detected after washing.	Increase in the CaP/collagen ratio, suggesting mineral deposition; carbonate content unchanged.	CHX showed low initial alteration of dentin but a favorable remineralization trend after ageing. Well-crystallized carbonated apatite formed after remineralization.
EDTA	Only minor changes were observed.	Increase in CaP/collagen ratio after ageing.	EDTA showed limited short-term dentin alteration but a favorable remineralization trend. Well-crystallized carbonated apatite formed after remineralization.
Citric acid	Marked dentin demineralization and adsorption of citrate ions.	Partial recovery of the CaP/collagen ratio, but not to the values observed in sound dentin.	Citric acid induced relevant initial demineralization and a less favorable remineralization trend, with the formation of a poorly carbonated and immature apatite layer.
NaClO	Partial alteration of the organic collagen phase.	No clear remineralization trend was observed after ageing.	NaClO mainly affected the organic dentin matrix and did not favor biomimetic remineralization.
Experimental irrigant	Strong demineralizing effect and adsorption of citrate ions onto collagen.	Reappearance of apatite bands and significant increase in the CaP/collagen ratio after ageing.	The experimental irrigant showed a remineralization trend thanks to citrate ions and surfactant adsorption on the surface.
Water control	No alteration.	No relevant remineralization trend.	Ageing in HBSS did not induce comparable mineral deposition, stressing the importance of irrigant pretreatment.

## Data Availability

Data are contained within the article or the Supplementary Material.

## Supplementary Materials

The following supporting information can be downloaded at https://www.mdpi.com/article/10.3390/biomimetics11070473/s1: Figure S1: Average IR spectra recorded on the dentin sample before and after treatment with the CHX solution for 10 min and washing. The spectrum of the dried solution is reported for comparison (Chl = bands of chlorhexidine; G = bands of gluconate ion). The spectra are normalized to the absorbance of the 1450 cm^−1^ band (chosen because of the negligible contribution of CHX in this spectral range). The bands assignable to collagen (Col) and apatite (Ap) are indicated together with those ascribable to CHX (*). Figure S2: Average IR spectra recorded on the dentin sample before and after treatment with the citric acid solution for 10 min and washing. The spectrum of the dried solution is reported for comparison. The spectra are normalized to the absorbance of the collagen band at about 1640 cm^−1^. The bands assignable to collagen (Col) and apatite (Ap) are indicated together with those ascribable to the effect of the irrigant (CA = citric acid; C^–^ = citrate). Figure S3: Average IR spectra recorded on the dentin sample before and after treatment with the citric acid solution for 10 min and washing. The spectra of commercial Ca(H_2_PO_4_)_2_ ∙ H_2_O and CaHPO_4_ ∙ 2H_2_O are reported for comparison. The bands that contribute HPO_4_^2−^ and H_2_PO_4_^−^ are indicated by a triangle. The spectral features assignable to collagen (Col) and apatite (Ap) are also indicated; see Figure S4 for the IR spectra of the dried experimental irrigant solution and 2-phenoxyethanol. In the spectrum of the irrigant, the bands ascribable to citric acid (CA) and 2-phenoxyethanol (P) are indicated. Figure S5: Average IR spectra recorded on the dentin sample before and after treatment with the experimental irrigant for 10 min and washing. The spectrum of a demineralized dentin slice (i.e., collagen) is reported for comparison. The spectra are normalized to the absorbance of the collagen band at about 1640 cm^−1^. The bands ascribable to the effect of the irrigant (C^–^ = citrate; S = surfactant) are indicated. Figure S6: Average IR spectra recorded on the dentin control sample (treatment with water) before and after ageing in HBSS for three months. The spectra are normalized to the absorbance of the collagen band at about 1640 cm^−1^. The bands assignable to collagen (Col) and apatite (Ap) are indicated.
