# Supplementary material for: Long-Term Influence of Endodontic Irrigants on In Vitro Dentin Biomimetic Remineralization
Source: Biomimetics (Basel). 2026 Jul 7;11(7):473. doi: 10.3390/biomimetics11070473 (PMC13406931; doi:10.3390/biomimetics11070473)
Supplement: Supplementary file 1 [file biomimetics-11-00473-s001.zip › biomimetics-4380770-supplementary.pdf]

biomimetics

Supplementary Material

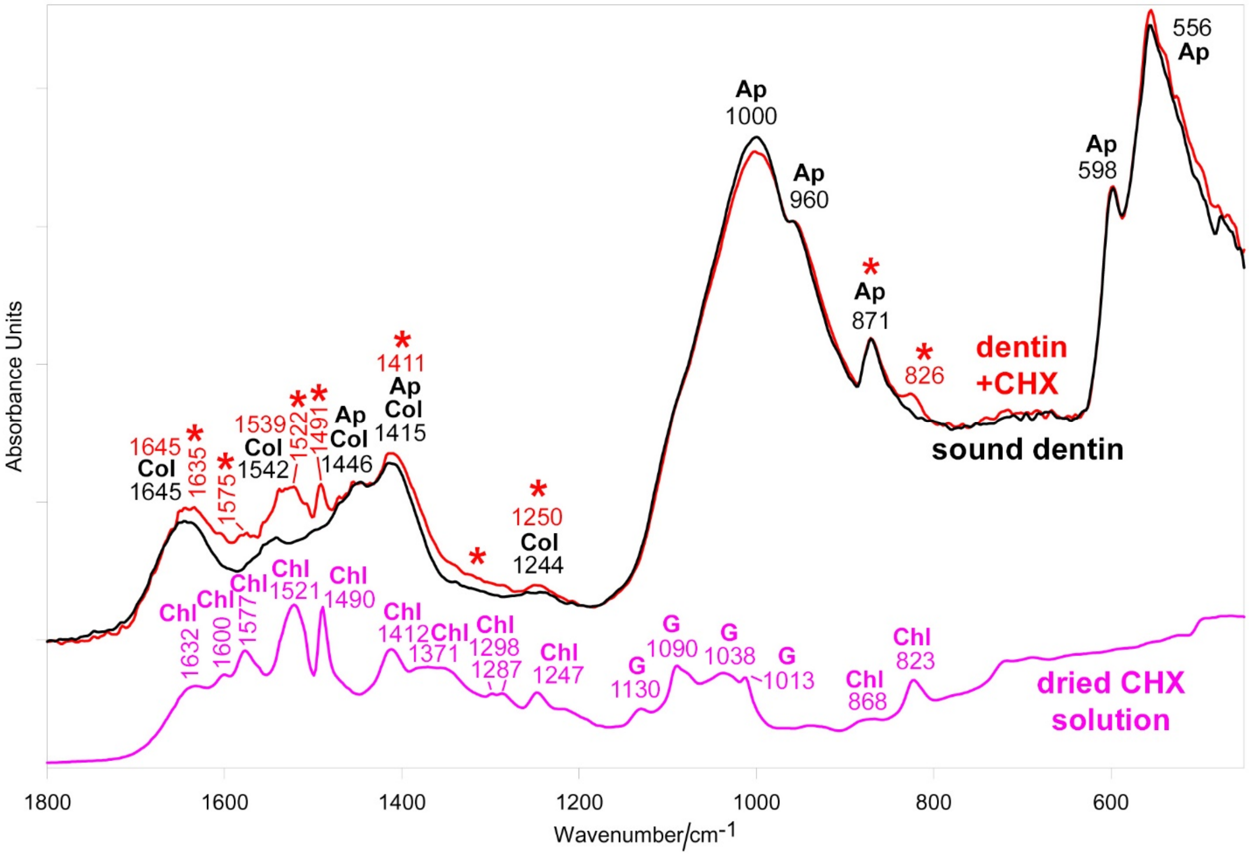

Figure S1. Average IR spectra recorded on the dentin sample before and after treatment with the CHX solution for 10 minutes and washing. The spectrum of the dried solution is reported for comparison (Chl = bands of chlorhexidine; G = bands of gluconate ion). The spectra are normalized to the absorbance of the 1450 cm^{-1} band (chosen because of the negligible contribution of CHX in this spectral range). The bands assignable to collagen (Col) and apatite (Ap) are indicated together with those ascribable to CHX (*).

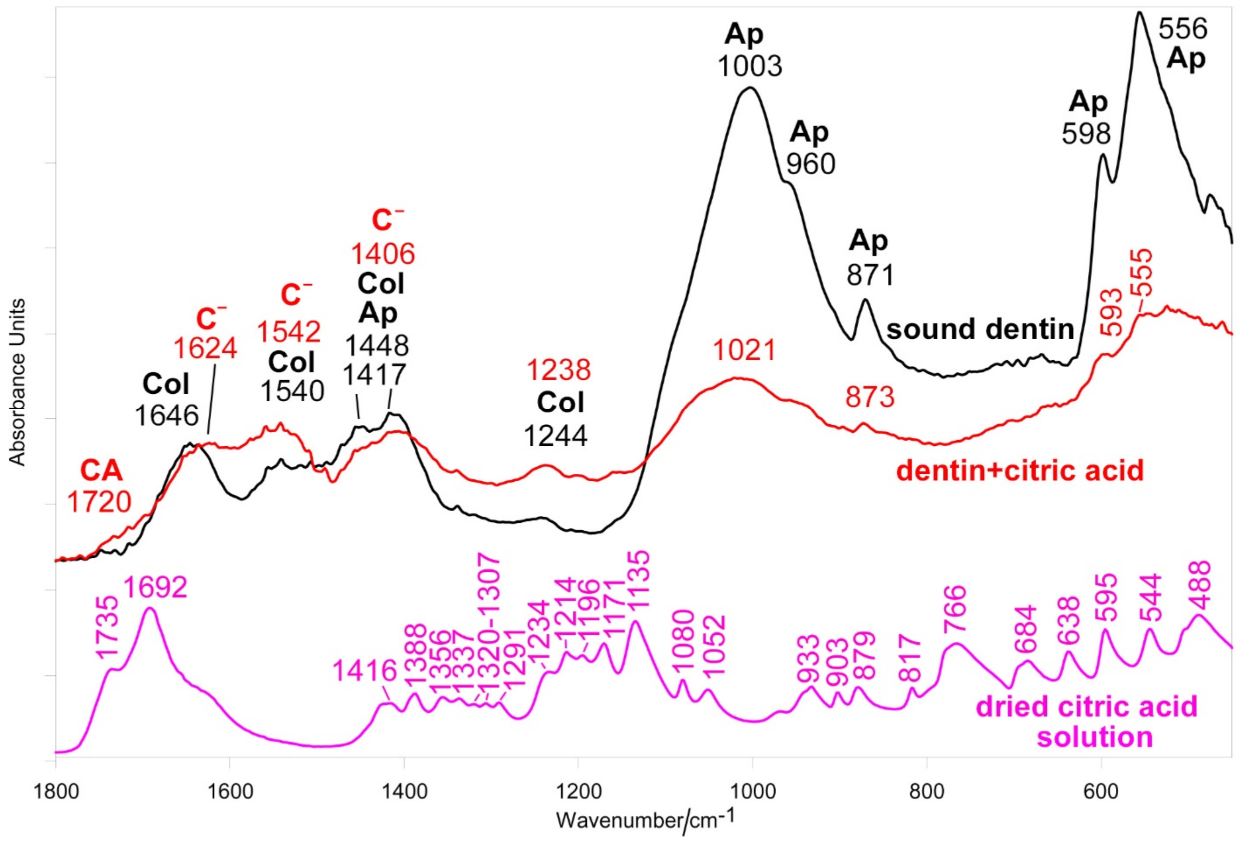

Figure S2. Average IR spectra recorded on the dentin sample before and after treatment with the citric acid solution for 10 minutes and washing. The spectrum of the dried solution is reported for comparison. The spectra are normalized to the absorbance of the collagen band at about 1640 cm^{-1}. The bands assignable to collagen (Col) and apatite (Ap) are indicated together with those ascribable to the effect of the irrigant (CA = citric acid; C^{-} = citrate).

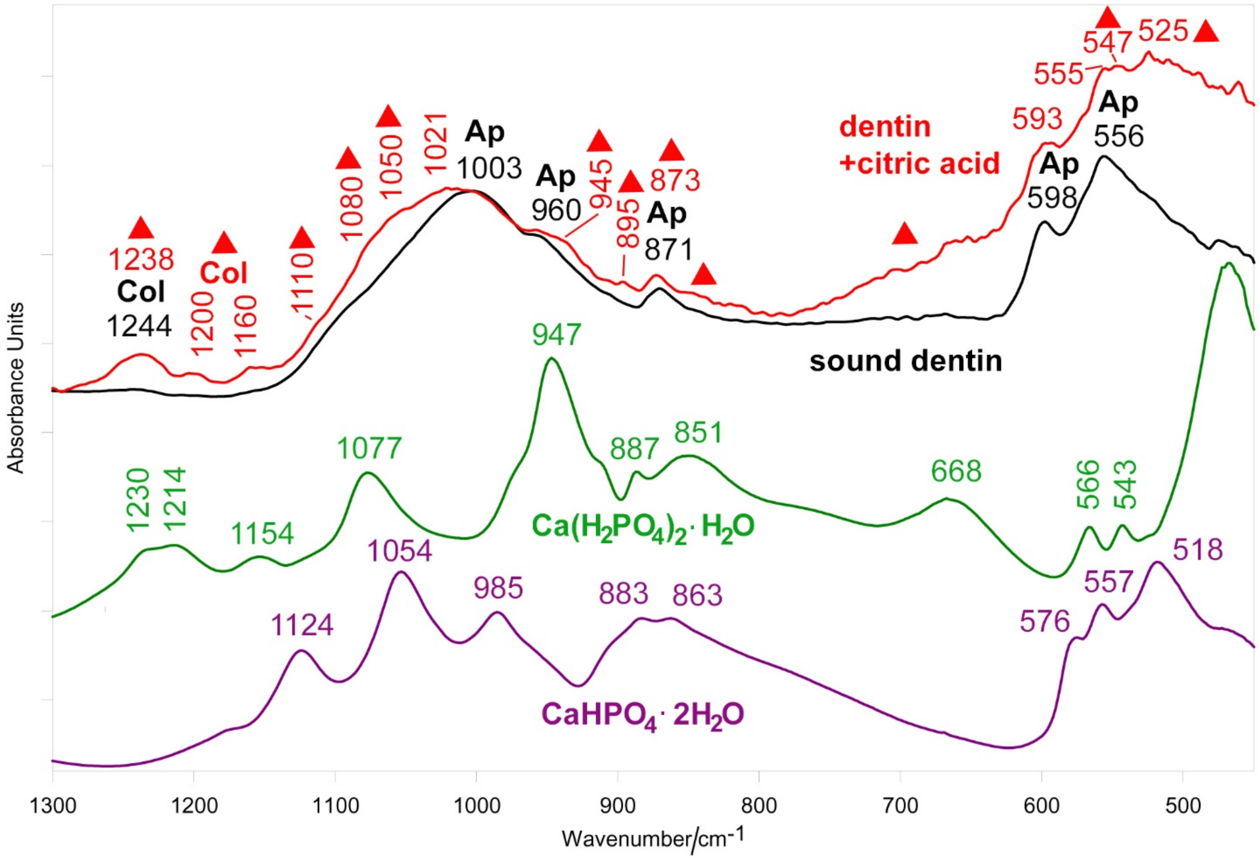

Figure S3. Average IR spectra recorded on the dentin sample before and after treatment with the citric acid solution for 10 minutes and washing. The spectra of commercial $Ca(H_2PO_4)_2 \cdot H_2O$ and $CaHPO_4 \cdot 2H_2O$ are reported for comparison. The bands with contributions from HPO_4^{2-} and H_2PO_4- ions are indicated by a triangle. The spectral features assignable to collagen (Col) and apatite (Ap) are indicated as well.

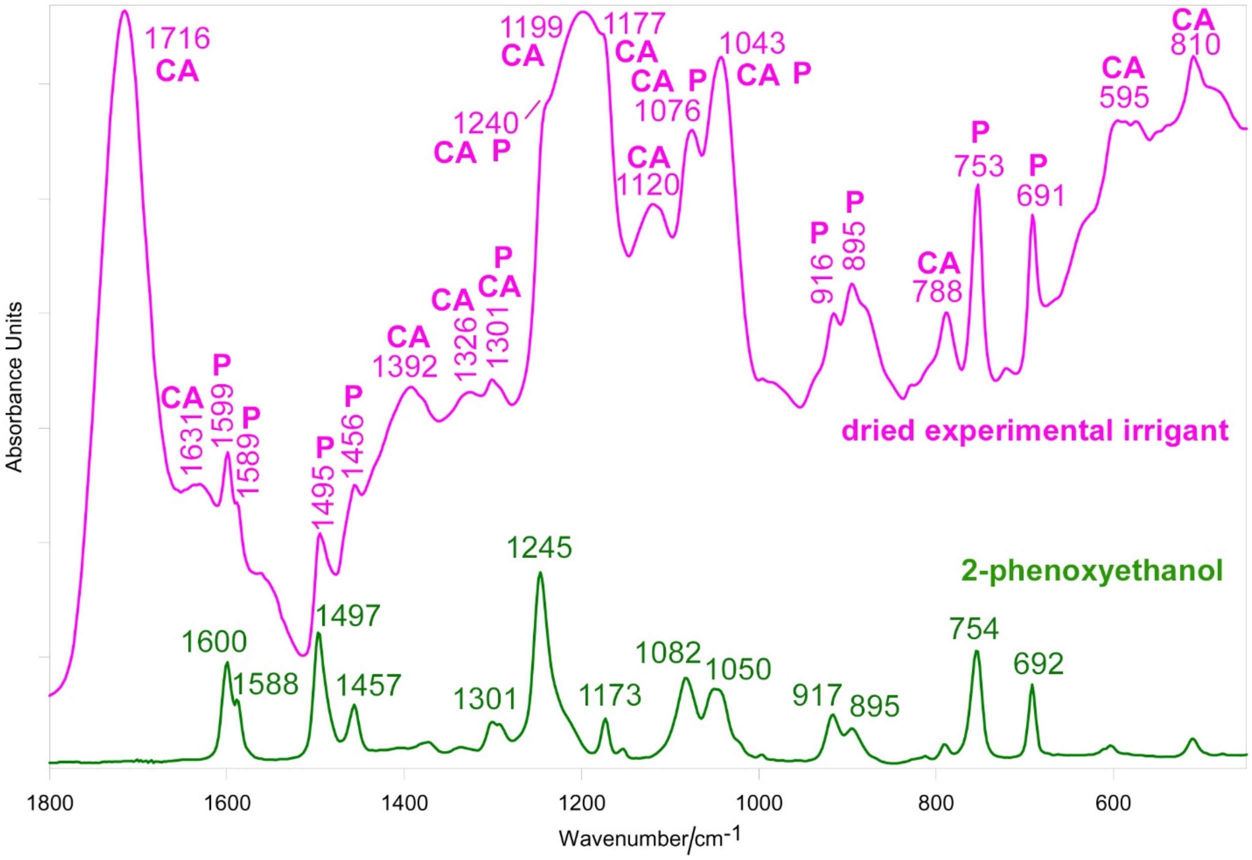

Figure S4. IR spectra of the dried experimental irrigant solution and 2-phenoxyethanol. In the spectrum of the irrigant, the bands ascribable to citric acid (CA) and 2-phenoxyethanol (P) are indicated.

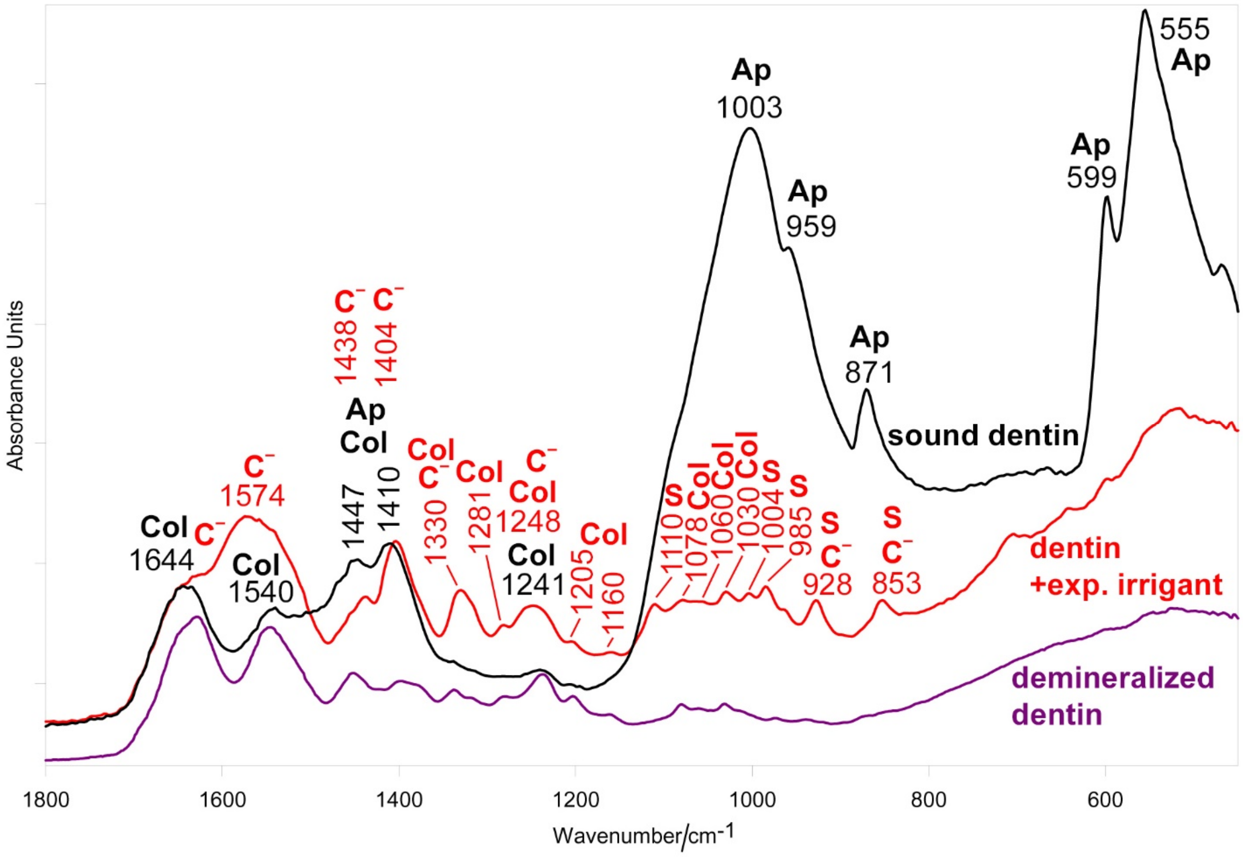

Figure S5. Average IR spectra recorded on the dentin sample before and after treatment with the experimental irrigant for 10 minutes and washing. The spectrum of a demineralized dentin slice (i.e., collagen) is reported for comparison. The spectra are normalized to the absorbance of the collagen band at about 1640 cm^{-1}. The bands ascribable to the effect of the irrigant (C^- = citrate; S = surfactant) are indicated.

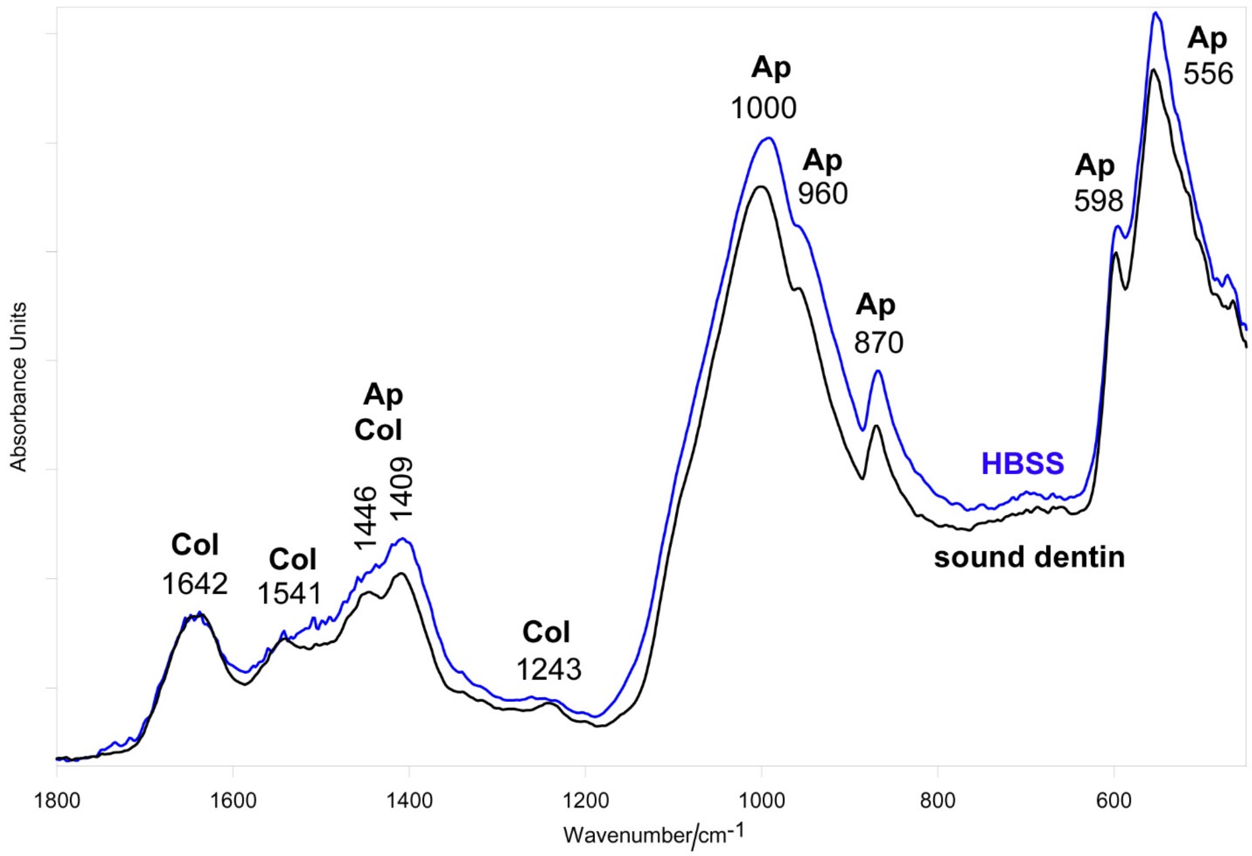

Figure S6. Average IR spectra recorded on the dentin control sample (treatment with water) before and after ageing in HBSS for three months. The spectra are normalized to the absorbance of the collagen band at about 1640 cm^{-1}. The bands assignable to collagen (Col) and apatite (Ap) are indicated.
